# Supplementary material for: Validation of a Multi-Residue Analysis Method for 287 Pesticides in Citrus Fruits Mandarin Orange and Grapefruit Using Liquid Chromatography–Tandem Mass Spectrometry
Source: Foods. 2022 Nov 4;11(21):3522. doi: 10.3390/foods11213522 (PMC9657228; doi:10.3390/foods11213522)
Supplement: Supplementary file 1 [file foods-11-03522-s001.zip › foods-2004786-supplementary.pdf]

Table S1. Method validation data for pesticide residue analysis in mandarin orange

No.	Compound name	LOQ (μg/kg)	R^2	Linear range (μg/kg)	Low			High			Matrix effect %
					Fortification (μg/kg)	Recovery %	RSD %	Fortification (μg/kg)	Recovery %	RSD %	
1	2,3,5-trimethacarb	1	0.9997	0.5–50	1	84.6	15.6	50	95.8	2.4	−1.41
2	3,4,5-trimethacarb	2.5	0.9997	0.5–50	2.5	87.8	3.5	50	99.3	0.4	5.56
3	Abamectin	2.5	0.9896	0.5–50	2.5	102.7	15.6	50	95.6	10.8	−9.21
4	Acetamiprid	1	0.9988	0.5–50	1	104.5	18	50	102.3	1.4	−31.56
5	Alachlor	1	0.9996	2–50	1	104	14.2	50	97.1	1	−4.16
6	Aldicarb sulfoxide	1	0.999	0.5–50	1	80.2	7.1	50	84.4	4.5	−7.79
7	Alidcarb sulfone	2.5	0.9998	1–50	2.5	97.9	1.7	50	91.1	2	−13.27
8	Ametoctradin	2.5	0.999	0.5–50	2.5	86.1	3.9	50	98.6	0.1	3.79
9	Ametryn	1	0.9996	2–50	1	85.8	11.5	50	101.3	7.2	3.79
10	Anilofos	2.5	0.999	0.5–50	2.5	79	10.8	50	100.6	1.8	−3.73
11	Aramite	1	0.9992	0.5–50	1	86.3	7	50	97.6	2.8	−3.98
12	Aspon	2.5	0.9999	0.5–50	2.5	94.3	6.3	50	95.6	0.8	−2.36
13	Atrazine	1	0.9994	0.5–50	1	79.7	15	50	101.6	0.4	4.88
14	Azaconazole	2.5	0.9994	0.5–50	2.5	107.6	7.2	50	99.5	3.1	15.29
15	Azamethiophos	2.5	0.9987	0.5–50	2.5	84.6	5.4	50	98.7	2.7	−4.51
16	Azimsulfuron	2.5	0.9996	0.5–50	2.5	85.3	8.8	50	83.6	0.2	7.86
17	Azoxystrobin	2.5	0.9995	0.5–50	2.5	95.8	5.2	50	96.1	4.5	−4.37
18	Benalaxyl	5	0.9969	0.5–50	5	104.3	11	50	101.7	1.8	−4.86
19	Bendiocarb	5	0.9984	0.5–50	5	114.4	4.5	50	103.7	2.3	8.91
20	Benoxacor	2.5	0.9991	0.5–50	2.5	82.9	9	50	100	1.7	2.13
21	Bensulfuron-methyl	1	0.9985	0.5–50	1	110.3	10.4	50	93.3	2.4	−8.94
22	Bensulide	5	0.9945	0.5–50	5	98.3	17.8	50	100.5	6.3	−15.09
23	Benthiavalicarb-isopropyl	2.5	0.9998	0.5–50	2.5	111.1	2.9	50	97	2.7	−7.36
24	Benzobicyclon	2.5	0.9999	0.5–50	2.5	105.3	7	50	102.9	13.2	3.16
25	Benzoylprop-ethyl	2.5	0.9992	0.5–50	2.5	86.7	2.8	50	97.9	4.6	−12.92
26	Bitertanol	5	0.9934	0.5–50	5	113.3	12.4	50	80.7	2.4	5.95
27	Bixafen	2.5	0.9998	0.5–50	2.5	98.7	5.5	50	92.8	1.4	3.15
28	Boscalid	2.5	1	0.5–50	2.5	107.8	7.4	50	90.5	4.2	1.79
29	Broflanilide	1	0.9978	0.5–50	1	95.3	17.3	50	86.2	3.1	−15.07
30	Broflanilide_DM-8007	5	0.9933	0.5–50	5	103.1	7.7	50	107	0.1	−0.93
31	Broflanilide_S(PFP-OH)-8007	5	0.997	0.5–50	5	112.3	13.9	50	90.9	4.5	21.66
32	Bromacil	2.5	0.9992	0.5–50	2.5	85	5.6	50	98.4	0.8	−3.17
33	Bromobutide	1	0.9996	0.5–50	1	80.9	16.3	50	98.7	2.4	−2.9
34	Bupirimate	2.5	0.9969	0.5–50	2.5	91.4	7.2	50	100	1.4	0.75
35	Buprofezin	2.5	0.9995	0.5–50	2.5	104.8	0.2	50	96.7	1.6	−10.06
36	Cadusafos	5	0.9958	0.5–50	5	99.3	0.9	50	100.5	1.2	2.33
37	Carbaryl	5	0.9953	0.5–50	5	86	8.1	50	101.5	2.7	6.38
38	Carbetamide	2.5	0.9998	0.5–50	2.5	105.6	7.5	50	99.6	2.2	−7.68

39	Carbofuran	2.5	0.9993	0.5–50	2.5	87.7	3.8	50	98.1	0.5	−18.41
40	Carboxin	2.5	0.9996	0.5–50	2.5	97.7	4.1	50	91.5	4.1	6.59
41	Carfentrazone-ethyl	2.5	0.9999	0.5–50	2.5	100.9	6.4	50	89.3	1.8	3.35
42	Carpropamid	2.5	0.9963	0.5–50	2.5	115.2	4.2	50	99.4	1.7	−4.73
43	Chlorantraniliprole	2.5	0.9981	0.5–50	2.5	97.3	7.4	50	101.1	2.4	1.1
44	Chlorbenzuron	5	0.9983	0.5–50	5	93.7	3.3	50	106.8	7.1	−5.59
45	Chlorfenvinphos	1	0.9993	0.5–50	1	84.1	12	50	98.9	2	−7.37
46	Chlorimuron-ethyl	1	0.9982	0.5–50	1	83.9	18.7	50	86.5	2.4	12.26
47	Chlorotoluron	5	0.9982	0.5–50	5	105	8.4	50	101.4	1	−13.86
48	Chloroxuron	1	0.9995	0.5–50	1	103.5	3	50	92.2	2.5	−5.73
49	Chlorpyrifos	2.5	0.9992	0.5–50	2.5	112.6	5.9	50	93.2	0.2	2.68
50	Chlorpyrifos-methyl	2.5	0.9985	0.5–50	2.5	87.9	14.2	50	98.5	2.4	−11.14
51	Chromafenozide	2.5	0.9964	0.5–50	2.5	118.1	9.2	50	106.1	8.2	−8.2
52	Clethodim	10	0.9961	1–50	10	99	8.6	50	96.6	1.1	−44.98
53	Clofentezine	2.5	0.9998	0.5–50	2.5	82.1	11.7	50	96.8	1.1	3.08
54	Clomazone	1	1	0.5–50	1	104.4	13.5	50	96	2.3	16.55
55	Clothianidin	2.5	0.9995	0.5–50	2.5	91.5	19.6	50	91.9	4.9	−13.05
56	Coumaphos	2.5	0.9967	0.5–50	2.5	82	7.5	50	98.8	3.2	−8.47
57	Crufomate	5	0.9934	0.5–50	5	94	10.3	50	104.2	2.5	−1.83
58	Cyanazine	2.5	0.9974	0.5–50	2.5	110	3.4	50	94.5	2.3	13.19
59	Cyazofamid	2.5	0.9965	0.5–50	2.5	100.2	10.4	50	106.1	0.9	−5.96
60	Cyclosulfamuron	2.5	0.9987	0.5–50	2.5	95.9	10.8	50	92.4	2.1	4.93
61	Cyflufenamid	2.5	0.9998	0.5–50	2.5	109.9	14.2	50	102.9	2.2	−2.34
62	Cyprazine	2.5	0.9989	0.5–50	2.5	73.7	14	50	104.3	3.4	9.32
63	Cyprodinil	5	0.9988	2–50	5	97.8	10.9	50	98.2	1.9	−2.83
64	Daimuron	2.5	0.999	0.5–50	2.5	114.3	1.5	50	95.9	3.8	0.49
65	Demeton-O	1	0.9991	0.5–50	1	114.7	1.8	50	96.2	3.5	18.84
66	Demeton-S	1	0.9975	0.5–50	1	77.1	4.2	50	102.4	0.7	−10.75
67	Demeton-S-methyl-sulfone	1	0.9998	0.5–50	1	114.6	15.4	50	93.7	1.3	−2.08
68	Desmetryn	2.5	0.9996	0.5–50	2.5	110.3	3.5	50	96.8	1.9	10.19
69	Diazinon	1	0.9998	0.5–50	1	86.8	2.7	50	101.4	2.9	−6.45
70	Dichlorvos	10	0.9989	0.5–50	10	84	13	50	103.7	4.3	0.46
71	Diclobutrazol	2.5	0.9999	0.5–50	2.5	97.4	19.1	50	109.5	1.1	−5.35
72	Dicrotophos	2.5	0.9971	0.5–50	2.5	77.5	14.7	50	101.4	1.5	−14.18
73	Diethatyl-ethyl	2.5	0.9979	0.5–50	2.5	82.5	3.3	50	101.2	1	−2.33
74	Diethofencarb	1	0.9994	0.5–50	1	85.2	4.9	50	96.2	0.6	1.07
75	Difenoconazole	1	0.9999	0.5–50	1	99	12.5	50	100.6	1.7	3.73
76	Diflubenzuron	2.5	0.9987	0.5–50	2.5	84.9	4.5	50	100.4	1.5	6.24
77	Diflufenican	2.5	0.9998	0.5–50	2.5	110.8	6.8	50	98.3	2.4	−0.39
78	Dimethachlor	1	0.9999	0.5–50	1	101.3	9.6	50	98.2	0.9	1.16
79	Dimethametryn	5	0.9927	0.5–50	5	92.9	11.3	50	103.1	2.3	−0.14
80	Dimethenamide	2.5	0.9942	0.5–50	2.5	71.3	20	50	99.7	4.2	−3.61
81	Dimethoate	1	0.9921	0.5–50	1	107.4	7.2	50	97	1.2	−39.62
82	Diniconazole	2.5	0.9998	0.5–50	2.5	70.4	5.9	50	95.1	3.3	1.92
83	Dinotefuran	2.5	0.9989	1–50	2.5	108	16.4	50	86	2	−19.33
84	Diphenamid	2.5	0.9927	0.5–50	2.5	113.9	3.1	50	82.8	1.8	2.95
85	Dithiopyr	1	0.9999	0.5–50	1	118.4	14.4	50	98.4	3.5	−2.89

86	Diuron	1	0.9993	0.5–50	1	101.8	13.9	50	99.4	0.8	19.87
87	Edifenphos	2.5	0.9941	0.5–50	2.5	70.8	7.5	50	98.5	5.9	−6.49
88	Epoxiconazole	5	0.997	0.5–50	5	89.6	6.1	50	97.9	3.5	−3.01
89	Esprocarb	2.5	0.999	0.5–50	2.5	93.7	3.5	50	99.9	2.2	12.57
90	Etaconazole	1	0.9996	0.5–50	1	99.4	2.1	50	95.2	4.9	0.14
91	Ethaboxam	2.5	0.9992	0.5–50	2.5	108.5	4.6	50	95.6	1.1	−6.55
92	Ethiofencarb	1	0.9997	0.5–50	1	114	15.5	50	98.4	1.1	4.8
93	Ethirimol	2.5	0.9999	0.5–50	2.5	97.8	14.1	50	93.1	1.8	−4.27
94	Ethoprophos	2.5	0.9984	0.5–50	2.5	81.2	5.6	50	94.6	3.3	5.48
95	Etofenprox	5	0.9995	0.5–50	5	108.6	2.7	50	93.7	1.8	2.34
96	Etoxazole	1	0.9993	0.5–50	1	102.8	4.9	50	79.1	1	−6.84
97	Etrimfos	2.5	0.9985	0.5–50	2.5	86.2	3.1	50	97.6	3.9	−6.59
98	Famoxadone	2.5	0.9963	0.5–50	2.5	100	8.9	50	84.3	0.7	19.81
99	Fenamiphos	2.5	0.9983	0.5–50	2.5	77.9	5.7	50	104.4	3.8	−0.86
100	Fenamiphos sulfone	2.5	0.9994	0.5–50	2.5	100.5	11.6	50	105.3	4	15.42
101	Fenamiphos sulfoxide	1	0.9998	0.5–50	1	79.8	14.1	50	93.9	0.7	−0.79
102	Fenarimol	1	0.997	0.5–50	1	105.5	13.2	50	93.9	2.3	−4.77
103	Fenbuconazole	2.5	0.9985	1–50	2.5	94.9	14.2	50	94.8	5	−9.2
104	Fenfuram	2.5	0.9985	0.5–50	2.5	78.6	13.6	50	102.3	1.1	−1.78
105	Fenobucarb	2.5	0.9968	0.5–50	2.5	90.8	8.1	50	109.9	3.8	10.87
106	Fenothiocarb	2.5	0.9993	0.5–50	2.5	87.5	4.1	50	103.2	3.6	−7.16
107	Fenoxanil	1	0.9996	0.5–50	1	94.2	4.7	50	101.8	3.3	7.85
108	Fenoxycarb	1	0.9989	0.5–50	1	105.8	14.7	50	92.9	1.9	−9.08
109	Fenpropimorph	2.5	0.9985	0.5–50	2.5	79.4	7.6	50	99	3.8	7.03
110	Fenpyroximate	1	0.9999	0.5–50	1	91.2	4.2	50	95	0.6	−0.31
111	Fenthion	2.5	0.9984	0.5–50	2.5	84.5	10.7	50	102	3.4	−10.04
112	Fenthion oxon	1	0.9995	0.5–50	1	98.6	5.7	50	102.2	0.5	−5.16
113	Fenthion oxon sulfone	1	0.9988	0.5–50	1	98.6	11.7	50	97.7	1.3	−12.94
114	Fenthion oxon sulfoxide	2.5	0.9998	0.5–50	2.5	99	16	50	94.2	2.9	−31.85
115	Fenthion sulfoxide	1	0.9994	0.5–50	1	74.7	17.4	50	101.5	2.1	−1.1
116	Fenthion-sulfone	5	0.9988	1–50	5	92	7	50	86.4	6	−3.08
117	Ferimzone	2.5	0.9983	1–50	2.5	77.5	6.4	50	104.1	2.3	−2.35
118	Fipronil	2.5	0.9973	0.5–50	2.5	112.7	16.5	50	99.7	0.5	−1.12
119	Fipronil-sulfone	2.5	0.999	0.5–50	2.5	109.5	2	50	99.9	1.7	−6.1
120	Flamprop-isopropyl	1	0.9993	0.5–50	1	88.9	10.5	50	98.6	1.9	−13.69
121	Flonicamid	1	0.9999	0.5–50	1	111.2	4.3	50	88.6	2.9	1.52
122	Fluazinam	1	0.9999	0.5–50	1	116.3	1.7	50	91.7	1.9	−6.42
123	Flucetosulfuron	2.5	0.9978	0.5–50	2.5	84.2	9.8	50	82.4	0.7	−0.16
124	Fludioxonil	2.5	0.9987	0.5–50	2.5	109.7	5.2	50	96.3	0.5	−9.96
125	Flufenacet	1	0.9996	0.5–50	1	82.1	2.2	50	100.6	4.2	−11.78
126	Flufenoxuron	1	0.9997	0.5–50	1	91	5.5	50	95.4	1.5	−1.11
127	Fluometuron	5	0.999	2–50	5	108.2	4.1	50	95.3	5	1.63
128	Fluopicolide	1	0.9994	0.5–50	1	104.4	2.4	50	96.8	1	−5.46
129	Fluopyram	1	0.9992	0.5–50	1	101	4.3	50	95.1	0.5	10.26
130	Flupyradifuron	1	0.9989	0.5–50	1	81.4	9.9	50	105.7	5.5	−18.47
131	Fluquinconazole	2.5	0.9998	0.5–50	2.5	114.9	15	50	96.3	5.2	−5.63
132	Fluridone	5	0.9976	0.5–50	5	100.7	2.1	50	105.2	1.2	−13.84

133	Flurochloridone	2.5	0.9994	0.5–50	2.5	111.2	12.3	50	103.8	3.2	3.98
134	Flurtamone	2.5	0.9959	0.5–50	2.5	71.6	5.7	50	103	0.6	−4.09
135	Flusilazole	1	0.9996	0.5–50	1	100.3	6.8	50	93.9	1.2	−5.78
136	Fluthiacet-methyl	2.5	0.9975	0.5–50	2.5	113.8	6.5	50	90	3.6	7.48
137	Flutianil	1	0.9987	0.5–50	1	111.2	2.6	50	96.6	1	−2.08
138	Flutolanil	2.5	0.9985	0.5–50	2.5	80.5	7	50	96	2.7	44.13
139	Flutriafol	2.5	0.9923	0.5–50	2.5	109.8	7	50	96.1	5	−12.44
140	Fluxapyroxad	2.5	0.9975	0.5–50	2.5	96.7	10.6	50	98.7	2.4	−7.98
141	Fosthiazate	1	1	0.5–50	1	101.8	4.6	50	97.7	1	−10.18
142	Furathiocarb	1	0.9998	0.5–50	1	114.7	9.9	50	96	1.7	18.79
143	Heptenophos	1	0.999	2–50	1	80.3	3.3	50	98	0.8	7.96
144	Hexaflumuron	5	0.9972	0.5–50	5	102.4	1.5	50	104.1	3	−5.76
145	Hexazinone	2.5	0.993	0.5–50	2.5	73.1	0.7	50	102.7	2.2	1.33
146	Hexythiazox	1	0.9998	0.5–50	1	115.6	9.7	50	93	1.3	2.88
147	Imibenconazole	2.5	0.9973	1–50	2.5	119.9	11.2	50	94.4	0.6	1.34
148	Imicyafos	2.5	0.9972	0.5–50	2.5	99.2	7.5	50	92.1	4.4	−1.65
149	Imidacloprid	1	0.9993	0.5–50	1	88.4	7	50	93.6	3.8	20.06
150	Inabenfide	1	0.9997	0.5–50	1	108.9	14.7	50	95.7	2.8	−0.72
151	Indanofan	2.5	0.9995	0.5–50	2.5	96	19.5	50	96.7	2.9	−7.64
152	Ipconazole	2.5	0.9998	0.5–50	2.5	101.7	5.7	50	90.3	0.8	−0.38
153	Iprobenfos	1	0.9983	2–50	1	83.1	10.4	50	98.6	2.5	−17.43
154	Iprovalicarb	2.5	0.9923	0.5–50	2.5	75.8	2.8	50	91.9	0.9	−2.95
155	Isoprocarb	5	0.9988	1–50	5	110.2	1.4	50	96.7	1.1	9.83
156	Isoprothiolane	1	0.9944	0.5–50	1	95	14.2	50	103	2.6	−15.24
157	Isopyrazam	2.5	0.9995	0.5–50	2.5	92.2	5.8	50	98.4	1.5	2.21
158	Isoxaben	5	0.9939	0.5–50	5	101.8	4.4	50	105.7	4.6	−8.26
159	Lenacil	5	0.9975	2–50	5	112.6	3.4	50	98.3	0.9	16.74
160	Linuron	1	0.9995	0.5–50	1	102.7	16.4	50	95.7	3.2	−4.74
161	Lufenuron	2.5	0.9981	0.5–50	2.5	92.2	15.9	50	89.2	0.8	−3.58
162	Malaoxon	1	0.999	0.5–50	1	116.1	5.3	50	98	1.5	5.54
163	Mandipropamid	2.5	0.9981	0.5–50	2.5	76.9	5.1	50	101.8	4.7	−0.86
164	Mecarbam	1	0.9988	0.5–50	1	71.8	11.8	50	103.8	3.6	0.8
165	Mefenacet	1	0.9977	0.5–50	1	107.5	8.2	50	98.7	3.7	−9.83
166	Mepanipyrim	2.5	0.9991	0.5–50	2.5	101.1	2.2	50	99.9	4	−3.67
167	Mephosfolan	1	0.9978	0.5–50	1	87.4	4.5	50	104.5	0.8	−6.58
168	Mepronil	2.5	0.9978	0.5–50	2.5	76.4	11.5	50	98.8	1.6	3.2
169	Metaflumizone (1)	5	0.9989	0.5–50	5	84.4	9.3	50	92.8	2.6	−10.03
	Metaflumizone (2)	2.5	0.9992	0.5–50	2.5	80.2	6.5	50	88.8	0.4	−10.03
170	Metalaxyl	2.5	0.9976	0.5–50	2.5	92.2	8.2	50	97.4	2	−0.14
171	Metamifop	2.5	0.9981	0.5–50	2.5	79.6	12.7	50	96.2	0.5	−9.92
172	Metconazole	2.5	0.9989	0.5–50	2.5	113.9	6	50	92.7	1.2	−3.82
173	Methabenzthiazuron	5	0.998	0.5–50	5	112.7	13.4	50	99	0.4	−22.21
174	Methiocarb-sulfone	2.5	0.9985	2–50	2.5	108.9	7.8	50	98.8	0.7	−39.35
175	Methiocarb-sulfoxide	1	0.9991	0.5–50	1	82.5	7	50	91	2	5.18
176	Methomyl	2.5	0.9999	0.5–50	2.5	91.7	10.5	50	95.6	3.9	−33.06
177	Methoprotryne	2.5	0.9982	0.5–50	2.5	83.1	9.5	50	98.3	0.1	−0.28
178	Metobromuron	1	0.9998	0.5–50	1	102.4	9	50	99.9	2.1	0.75

179	Metominostrboin (Z)	1	0.9998	0.5–50	1	106.9	2.9	50	99.7	2.6	1.8
180	Metominostrobin (E)	2.5	0.9975	0.5–50	2.5	78	11	50	100.5	0.3	−4.97
181	Metrafenon	5	0.9996	0.5–50	5	93.9	1.5	50	96.1	3.2	2.78
182	Mevinphos (1)	2.5	0.9993	0.5–50	2.5	105.1	7.6	50	97.9	1.8	−17.41
	Mevinphos (2)	1	0.9998	0.5–50	1	90.3	9.6	50	96.6	3.1	−17.41
183	Monocrotophos	1	0.9997	0.5–50	1	108.9	10	50	97.3	1.3	1.37
184	Myclobutanil	2.5	0.9992	2–50	2.5	116.5	13.6	50	92.5	1.2	−26.58
185	Napropamide	2.5	0.998	0.5–50	2.5	89.3	6.9	50	98.6	2.3	1.53
186	Neburon	1	0.9988	0.5–50	1	87.4	11.4	50	98.1	1.6	−7.16
187	Novaluron	2.5	0.9949	0.5–50	2.5	80.5	7.5	50	108.5	3.6	−0.85
188	Nuarimol	1	0.9996	1–50	1	86.5	7.2	50	95.6	2.4	−2.61
189	Ofurace	2.5	0.9991	1–50	2.5	111.9	4.3	50	95.1	3	0.54
190	Omethoate	2.5	0.9994	1–50	2.5	83.2	12.3	50	93.5	4.1	−18.64
191	Oxadiazon	2.5	0.9997	2–50	2.5	116	1.2	50	87.8	3.3	−9.32
192	Oxamyl	2.5	0.9999	0.5–50	2.5	105.3	2.8	50	94.6	2.6	−13.97
193	Oxaziclomefone	2.5	0.9984	0.5–50	2.5	85.5	1	50	97.2	2.2	5.47
194	Penconazole	2.5	0.9977	2–50	2.5	112.6	16	50	94.8	5.7	−12.81
195	Pencycuron	1	0.9988	0.5–50	1	97	2.6	50	84.4	4.5	−4.42
196	Phenthoate	2.5	0.9993	2–50	2.5	105.3	11.7	50	96.1	1.4	−7.1
197	Phosalone	1	0.9999	0.5–50	1	112.6	18.5	50	90.6	2.5	−3.8
198	Phosfolan	5	0.9988	0.5–50	5	106.4	7.5	50	101	2.2	−3.61
199	Phosmet-oxon	2.5	0.9988	0.5–50	2.5	86.6	11.5	50	100	2.6	−7.14
200	Phosphamidon	1	0.9998	0.5–50	1	100.6	3.8	50	98	1.2	17.61
201	Phoxim	2.5	0.9977	2–50	2.5	84.4	10.1	50	99.4	2.6	−5.28
202	Picolinafen	1	1	0.5–50	1	104.1	8.1	50	97.3	0.9	0.8
203	Pinoxaden	5	0.9933	0.5–50	5	90.9	16.6	50	102	1.7	−0.05
204	Piperonyl butoxide	2.5	0.9987	0.5–50	2.5	90.3	5.5	50	99.1	2.8	3.12
205	Piperophos	2.5	0.9997	0.5–50	2.5	105.8	4.4	50	99.5	0.8	−1.3
206	Pirimicarb	2.5	0.9994	0.5–50	2.5	88.2	0.1	50	100.4	0.3	−1.25
207	Pirimicarb-desmethyl	1	0.999	0.5–50	1	116.9	0.9	50	95.7	2.7	−22.24
208	Pirimiphos-ethyl	1	0.9992	0.5–50	1	87.5	6.5	50	81.3	0.6	−3.01
209	Pirimiphos-methyl	2.5	0.9985	0.5–50	2.5	88.4	3.3	50	102	1.6	−7.23
210	Prochloraz	1	0.9998	2–50	1	90.3	6.2	50	95.5	3	−4.43
211	Profenofos	1	0.9999	0.5–50	1	89.4	1.3	50	97.5	0.9	3.8
212	Promecarb	2.5	0.9984	0.5–50	2.5	86	10.7	50	93.8	3.8	9.16
213	Prometryn	5	0.9963	0.5–50	5	95.4	3.9	50	98.4	4	−1.77
214	Pronamide (Propyzamide)	1	0.9989	0.5–50	1	89.7	16.2	50	96.2	4.8	−4.6
215	Propachlor	1	0.9997	0.5–50	1	88.6	17.3	50	98	0.9	10.74
216	Propamocarb	1	0.9998	0.5–50	1	76.1	3.6	50	88.3	0.7	−13.36
217	Propanil	1	0.9996	2–50	1	113.7	18.7	50	97.7	0.1	−6.43
218	Propaquizafop	1	0.9997	0.5–50	1	84.3	13.7	50	95.6	0.7	4.02
219	Propargite	2.5	0.9998	0.5–50	2.5	107.4	2.2	50	96.1	1.3	−5.35
220	Propazine	2.5	0.9991	0.5–50	2.5	92.9	7.8	50	96.9	2.5	11.83
221	Propiconazole	5	0.9991	2–50	5	105.8	11.9	50	97	1.8	−2.88
222	Propoxur	2.5	0.998	0.5–50	2.5	83.5	3.9	50	106	4.1	5.62
223	Proqunazid	1	0.9993	0.5–50	1	87.5	3.1	50	97.1	0.3	−3.91
224	Prothioconazole	2.5	0.9987	2–50	2.5	100.5	2.7	50	103.4	1.5	1.35

225	Prothioconazole-desthio	1	0.9977	2–50	1	75.6	16.2	50	111.4	1.1	−9.01
226	Pydiflumetofen	2.5	0.9988	0.5–50	2.5	97	6	50	101.4	2.6	2.26
227	Pyracarbolid	2.5	0.9987	0.5–50	2.5	77.4	15.3	50	100.7	0.8	−0.32
228	Pyraclofos	1	0.9992	0.5–50	1	88.5	10	50	101.5	1.1	−3.81
229	Pyraclonil	2.5	0.9998	0.5–50	2.5	116.1	3.8	50	99.2	0.7	−10.96
230	Pyraclostrobin	2.5	0.9967	0.5–50	2.5	71.5	17.2	50	100.2	1.5	−1.74
231	Pyraflufen-ethyl	10	0.991	2–50	10	108.6	3.1	50	96.7	1	−6.23
232	Pyraziflumid	5	0.9992	0.5–50	5	105	2.9	50	93.3	3.8	−4.95
233	Pyrazolate	1	0.9994	0.5–50	1	89.3	8.5	50	102.7	6.8	−4.52
234	Pyrazophos	2.5	0.9998	0.5–50	2.5	108.6	4.9	50	97.5	5.3	10.56
235	Pyributicarb	1	0.9999	0.5–50	1	74.3	7	50	105.8	3.1	0.01
236	Pyridaben	1	0.9998	0.5–50	1	86	3.7	50	94	3.5	−2.18
237	Pyridalyl	2.5	0.9994	2–50	2.5	104.3	4.4	50	99.6	2.1	15.72
238	Pyrifluquinazon	5	0.9908	0.5–50	5	80.6	5.7	50	92.8	0.8	1.12
239	Pyriftalid	1	0.9999	0.5–50	1	90.1	8.6	50	102	2.5	−4.54
240	Pyrimethanil	5	0.9981	2–50	5	106.5	7.3	50	93.3	0.7	−1.69
241	Pyrimidifen	2.5	0.9944	0.5–50	2.5	76.1	3.8	50	96.5	2.2	−1.03
242	Pyrimisulfan	1	0.9998	0.5–50	1	98	18.6	50	94.9	1.3	8.3
243	Pyriproxyfen	2.5	0.9959	0.5–50	2.5	76.4	8.8	50	98.4	0.3	−7.65
244	Pyroquilon	2.5	0.9995	2–50	2.5	115	4.2	50	102.3	3.8	−2.3
245	Quinalphos	2.5	0.9998	2–50	2.5	92.8	4.6	50	93.7	2.7	−11.3
246	Quizalofop-ethyl	2.5	0.9994	0.5–50	2.5	106.6	2.1	50	91.9	1.7	0.07
247	Simazine	10	0.9996	2–50	10	108.6	7.2	50	101.5	7.6	−10.92
248	Simetryn	1	0.9997	0.5–50	1	72.1	4.8	50	97	0.9	5.25
249	Spinetoram (J)	2.5	0.9999	0.5–50	2.5	89.5	7.7	50	98.4	1.3	−10.97
250	Spinetoram (L)	1	0.9996	0.5–50	1	101.3	10.8	50	88.6	2.5	−10
251	Spinosyn A	1	0.9999	2–50	1	103.1	13.9	50	98.2	0.9	−5
252	Spinosyn D	1	0.9998	1–50	1	105.5	12.5	50	88.4	0.9	2.37
253	Spirodiclofen	1	0.9999	0.5–50	1	95.8	5.7	50	91.7	1.1	12.06
254	Spirotetramat-ketohydroxy	5	0.9995	0.5–50	5	93.2	8.1	50	90.3	0.5	2.97
255	Spirotetramat-monohydroxy	2.5	0.9943	0.5–50	2.5	83.8	12.4	50	99.3	1.5	3.77
256	Spiroxamine	2.5	0.9988	0.5–50	2.5	88.8	2.1	50	98.6	1.6	−5.18
257	Sulfentrazone	2.5	0.9944	0.5–50	2.5	113.6	16	50	96	3.9	−6.23
258	Sulfotep	2.5	0.9962	0.5–50	2.5	73.9	13.8	50	99.6	0.6	−1.67
259	Sulfoxaflor	2.5	0.9995	0.5–50	2.5	92.1	8.3	50	99.7	1.2	17.05
260	Sulprofos	2.5	0.9997	0.5–50	2.5	111.4	4.8	50	97.2	0.7	−0.52
261	TCMTB	5	0.9967	1–50	5	115.7	2.4	50	97.4	0.3	4.54
262	Tebufenpyrad	1	0.9999	0.5–50	1	93.3	0.9	50	96.8	0.7	1.88
263	Tebuthiuron	5	0.9975	0.5–50	5	117.7	6.1	50	105.9	2.2	3.81
264	Tepraloxydim	5	0.9971	0.5–50	5	105.7	3.2	50	94.3	1.5	18.06
265	Terbuthylazine	1	0.9988	1–50	1	97	5.8	50	99.9	1.8	3.39
266	Terbutryn	5	0.9949	0.5–50	5	101.2	1.4	50	99.4	2.5	−3.13
267	Tetrachlorvinphos	2.5	0.9992	0.5–50	2.5	84.1	14.9	50	95.4	3.8	−0.4
268	Tetraconazole	1	0.9999	2–50	1	92	12.9	50	95.9	7.4	−4.65
269	Thenylchlor	1	0.999	0.5–50	1	97.2	13.5	50	101.4	2.6	−15.92
270	Thiabendazole	2.5	0.9999	0.5–50	2.5	94	12.2	50	93	0.5	−39.09
271	Thiacloprid	2.5	0.9955	0.5–50	2.5	72.7	4.2	50	97.6	1.5	−3.71

272	Thidiazuron	1	0.9997	0.5–50	1	109.6	1.2	50	90	1	−2.42
273	Thifluzamide	5	0.9966	0.5–50	5	99.7	9.5	50	103.2	4.4	−2.85
274	Thiobencarb	2.5	0.9998	2–50	2.5	101.3	13.7	50	102.2	3	−4.16
275	Thiodicarb	2.5	0.9998	0.5–50	2.5	114.8	0.8	50	91.4	2.8	66.19
276	Thionazin	2.5	0.9982	1–50	2.5	114.1	2	50	96.3	1.2	14.2
277	Tiadinil	2.5	0.9994	0.5–50	2.5	114	4	50	90.4	3.1	7.72
278	Tolfenpyrad	1	0.9999	0.5–50	1	105.5	6.3	50	91	1.7	3.72
279	Triadimefon	2.5	0.9984	0.5–50	2.5	116.9	7.2	50	93.5	0.5	−7.24
280	Triazophos	1	0.9987	0.5–50	1	102.8	2.5	50	80	5	−3.34
281	Tricyclazole	2.5	0.9971	0.5–50	2.5	71.9	3.6	50	98.5	2.5	−9.68
282	Trifloxystrobin	2.5	0.9995	0.5–50	2.5	94.2	5.5	50	102.2	2.6	17.4
283	Triflumizole	1	0.9998	0.5–50	1	113	2.5	50	94.6	1.7	−5.73
284	Triflumuron	2.5	0.9996	0.5–50	2.5	92.6	4	50	94.1	1.4	−7.37
285	Triticonazole	2.5	0.9989	0.5–50	2.5	115.6	13.1	50	94	3.1	−14.09
286	Vamidothion	1	0.9992	0.5–50	1	95	10.9	50	99.3	2	−24.23
287	Zoxamide	1	0.9992	0.5–50	1	101	5.7	50	89	2.6	8.95

Table S2. Method validation data for pesticide residue analysis in grapefruit

**No.**	**Compound name**	**LOQ (μg/kg)**	R^2	**Linear range (μg/kg)**	**Low**			**High**			**Matrix effect %**
					**Fortification (μg/kg)**	**Recovery %**	**RSD %**	**Fortification (μg/kg)**	**Recovery %**	**RSD %**	
1	2,3,5-trimethacarb	1	0.9998	0.5–50	1	81	3.6	50	102.8	8.3	−4.57
2	3,4,5-trimethacarb	1	0.9975	0.5–50	1	72	10.4	50	92.3	1.4	2.23
3	Abamectin	2.5	0.9916	1–50	2.5	113.3	5.1	50	98.3	11.4	−99.97
4	Acetamiprid	2.5	0.9993	0.5–50	2.5	112.1	1.7	50	87.9	0.8	−43.09
5	Alachlor	2.5	0.9994	0.5–50	2.5	88.9	6.7	50	93	5.7	−6.61
6	Aldicarb sulfoxide	2.5	0.9996	0.5–50	2.5	99.9	12.2	50	94.6	6.3	−51.04
7	Alidcarb sulfone	5	0.9998	0.5–50	5	113.2	13.1	50	95.7	4.5	−32.56
8	Ametoctradin	2.5	0.9972	0.5–50	2.5	76.4	4.6	50	91.5	5.6	−9.01
9	Ametryn	1	0.9997	0.5–50	1	93.3	14.9	50	91.4	3	0.51
10	Anilofos	2.5	0.9967	0.5–50	2.5	71.6	4.9	50	105.8	3.8	−0.89
11	Aramite	1	0.9987	0.5–50	1	77.7	14.3	50	98.2	5.1	−2.06
12	Aspon	2.5	0.999	0.5–50	2.5	109.4	4.8	50	99.2	8.1	−5.73
13	Atrazine	1	0.9999	0.5–50	1	91.7	4.7	50	97	3.6	2.23
14	Azaconazole	1	0.9992	0.5–50	1	100.3	13	50	98	1.4	2.47
15	Azamethiophos	2.5	0.9996	0.5–50	2.5	119.6	1.1	50	94.1	3.3	−28.64
16	Azimsulfuron	1	0.9993	0.5–50	1	78.2	7	50	81.3	9.3	10.08
17	Azoxystrobin	2.5	0.9978	0.5–50	2.5	79	2	50	97.8	7.3	−8.71
18	Benalaxyl	2.5	0.9988	0.5–50	2.5	77.1	8.2	50	92.3	3.4	−4.87
19	Bendiocarb	2.5	0.9985	0.5–50	2.5	110.7	9.2	50	88.7	2	−8.45

20	Benoxacor	2.5	0.9998	0.5–50	2.5	85.3	2.9	50	98.3	1.6	0.2
21	Bensulfuron-methyl	1	0.9997	0.5–50	1	78.1	11.5	50	93.2	8.5	14.03
22	Bensulide	2.5	0.9983	0.5–50	2.5	82.9	15.3	50	77.7	6.1	34.63
23	Benthiavalicarb-isopropyl	2.5	0.9994	0.5–50	2.5	88.5	5.3	50	97.7	1.7	−6.07
24	Benzobicyclon	2.5	0.996	0.5–50	2.5	72.1	0.4	50	95.3	3.4	10.91
25	Benzoylprop-ethyl	2.5	0.999	0.5–50	2.5	83.8	0	50	97.2	5.5	−5.31
26	Bitertanol	2.5	0.9945	0.5–50	2.5	107.3	0.2	50	89.7	5.7	11.13
27	Bixafen	2.5	0.9986	0.5–50	2.5	76.3	18.7	50	100.7	10.4	1.12
28	Boscalid	2.5	0.9996	0.5–50	2.5	97.3	13.2	50	98.4	1	−2.29
29	Broflanilide	5	0.9988	0.5–50	5	91.9	14.3	50	96.1	5.1	−11.12
30	Broflanilide_DM-8007	2.5	0.9997	0.5–50	2.5	70.3	7.3	50	94.7	9	12.98
31	Broflanilide_S(PFP-OH)-8007	5	0.9947	0.5–50	5	99	14	50	110.5	2.2	−45.72
32	Bromacil	5	0.9998	0.5–50	5	88.7	11.6	50	95.5	0	−27.44
33	Bromobutide	2.5	0.9988	0.5–50	2.5	106.5	6.2	50	89.2	5.7	0.21
34	Bupirimate	2.5	0.9985	0.5–50	2.5	87.7	14.9	50	93.7	0.5	−2.26
35	Buprofezin	1	0.9994	0.5–50	1	108	4.3	50	87	3.8	−5.74
36	Cadusafos	5	0.9978	0.5–50	5	90.6	6.6	50	97.3	1.7	3.84
37	Carbaryl	2.5	0.9993	0.5–50	2.5	94.8	8.9	50	109.6	13.5	2.25
38	Carbetamide	2.5	0.9998	0.5–50	2.5	101	5.1	50	100.2	5.8	−21.53
39	Carbofuran	1	0.9996	0.5–50	1	75.4	1.6	50	102.9	0.1	−39.5
40	Carboxin	5	0.9989	0.5–50	5	97.6	0.8	50	100.3	1.5	0.47
41	Carfentrazone-ethyl	2.5	0.9987	0.5–50	2.5	103.4	0.1	50	93.3	1.3	−6.44
42	Carpropamid	2.5	0.9998	0.5–50	2.5	111.9	7.8	50	88.9	4.7	−5.48
43	Chlorantraniliprole	5	0.9989	0.5–50	5	109.8	12.7	50	97.8	1.3	3.78
44	Chlorbenzuron	5	0.9971	0.5–50	5	80.7	0.1	50	96.8	6.2	−6.29
45	Chlorfenvinphos	2.5	0.9993	0.5–50	2.5	93.1	7.6	50	100.2	5.3	−8.35
46	Chlorimuron-ethyl	5	0.9996	0.5–50	5	88.7	8.9	50	83.7	9.9	10.05
47	Chlorotoluron	1	0.9995	0.5–50	1	117.6	13.9	50	86.4	9.7	−16.48
48	Chloroxuron	2.5	0.999	0.5–50	2.5	85.6	8.6	50	98.1	4.4	0.62
49	Chlorpyrifos	2.5	0.9998	0.5–50	2.5	87.8	4.2	50	93.6	3	−12.52
50	Chlorpyrifos-methyl	5	0.9988	0.5–50	5	101.4	6.9	50	101	0.1	−1.83
51	Chromafenozide	5	0.9996	0.5–50	5	93.2	15.6	50	74.6	11	−32.99
52	Clethodim	10	0.9992	0.5–50	10	86.9	0.1	50	94.8	0.1	−17.4
53	Clofentezine	2.5	0.999	0.5–50	2.5	79.9	3.1	50	88.4	1.8	−7
54	Clomazone	2.5	0.9991	0.5–50	2.5	87.1	11.4	50	98.9	2.3	−2.68
55	Clothianidin	2.5	0.9996	0.5–50	2.5	78.1	7.4	50	90.8	10.2	−18.09
56	Coumaphos	2.5	0.9987	0.5–50	2.5	82.1	5	50	95	4.4	−2.05
57	Crufomate	5	0.991	0.5–50	5	108	11.8	50	106	1.5	−1.52
58	Cyanazine	1	0.9999	0.5–50	1	107.7	7.8	50	94.9	0.2	−2.92
59	Cyazofamid	5	0.9913	0.5–50	5	118	14.1	50	119	2.6	−3.08
60	Cyclosulfamuron	1	0.9979	0.5–50	1	99.8	11	50	93.1	6.6	3.61
61	Cyflufenamid	5	0.9957	0.5–50	5	88.2	7.7	50	96.6	6.3	−7.49
62	Cyprazine	2.5	0.9988	0.5–50	2.5	86.4	10.1	50	99.1	6.9	−0.92
63	Cyprodinil	2.5	0.9996	0.5–50	2.5	86.5	0.5	50	97	3	−5.86
64	Daimuron	1	0.9995	0.5–50	1	112.4	4	50	103.8	5.5	−2.06
65	Demeton-O	2.5	0.9952	0.5–50	2.5	88.8	3.4	50	103.6	0	16.16
66	Demeton-S	5	0.9985	0.5–50	5	103.1	15	50	93.6	7.1	7.66

67	Demeton-S-methyl-sulfone	1	0.9992	0.5–50	1	98.8	4.2	50	93.3	1.4	−19.21
68	Desmetryn	1	0.9998	0.5–50	1	78	14.3	50	99.1	2.1	−7.07
69	Diazinon	1	0.9997	0.5–50	1	94.1	5.9	50	93.5	4.8	−4.1
70	Dichlorvos	10	0.9979	0.5–50	10	112.2	1.2	50	117.9	1.2	−7.64
71	Diclobutrazol	2.5	0.9983	0.5–50	2.5	117	10.3	50	105.6	8	−15.61
72	Dicrotophos	2.5	0.9994	0.5–50	2.5	110.8	1.3	50	92.6	2.5	4
73	Diethatyl-ethyl	1	0.9998	0.5–50	1	97	14.4	50	103.4	3.6	−15.05
74	Diethofencarb	2.5	0.9981	0.5–50	2.5	84.2	10	50	97.4	1.2	1.2
75	Difenoconazole	1	0.9999	0.5–50	1	87.9	13.2	50	97.5	1.7	5.11
76	Diflubenzuron	2.5	0.9983	0.5–50	2.5	95.4	5	50	91.4	3.9	−12.47
77	Diflufenican	1	0.9989	0.5–50	1	70.7	19.2	50	95.1	5.7	−4.56
78	Dimethachlor	1	0.9997	0.5–50	1	82.2	6.1	50	95.9	0.1	4.24
79	Dimethametryn	5	0.9919	0.5–50	5	93.4	16	50	103.4	7.9	−4.38
80	Dimethenamide	2.5	0.9986	0.5–50	2.5	82.5	6.3	50	96.3	3.1	−2.7
81	Dimethoate	1	0.9999	0.5–50	1	90.9	1.5	50	96.6	2.5	−40.98
82	Diniconazole	2.5	0.9978	0.5–50	2.5	73.9	9.7	50	92.7	6.5	3.06
83	Dinotefuran	2.5	0.9979	1–50	2.5	103	13.6	50	98.3	5.1	−53.6
84	Diphenamid	1	0.9938	0.5–50	1	107.2	1.8	50	99.5	3.5	5.11
85	Dithiopyr	2.5	0.9982	0.5–50	2.5	83	7.9	50	95.2	10.2	8.61
86	Diuron	1	0.9997	0.5–50	1	85.4	18.1	50	94.6	1.5	−5.86
87	Edifenphos	5	0.9977	1–50	5	92.1	1.1	50	91.3	2.7	−4.5
88	Epoxiconazole	1	0.9982	0.5–50	1	87.8	13.4	50	94.4	5.3	−7.26
89	Esprocarb	2.5	0.997	0.5–50	2.5	73.5	0.8	50	96.2	3.9	−3.51
90	Etaconazole	5	0.9988	1–50	5	102	3.4	50	96	5.8	−13.67
91	Ethaboxam	1	0.9996	0.5–50	1	90.4	7	50	97	5.2	−8.27
92	Ethiofencarb	1	0.9998	0.5–50	1	81.2	0.9	50	97	1.3	5.69
93	Ethirimol	2.5	0.9997	0.5–50	2.5	107	6.5	50	91	0.5	−9.35
94	Ethoprophos	1	0.9986	1–50	1	106.9	8.2	50	91.8	5.4	−9
95	Etofenprox	1	0.9957	0.5–50	1	107.4	14.3	50	98.4	5	−0.84
96	Etoxazole	5	0.9916	0.5–50	5	94	5.9	50	98	5.3	−12.18
97	Etrimfos	2.5	0.9993	1–50	2.5	91.1	1.9	50	98.3	1.8	−7.33
98	Famoxadone	10	0.9983	0.5–50	10	103	10.7	50	91.2	10.7	−13.05
99	Fenamiphos	2.5	0.9974	0.5–50	2.5	72	15.7	50	104.3	1.8	−0.26
100	Fenamiphos sulfone	2.5	0.9993	0.5–50	2.5	112.2	4.6	50	99.1	1.8	9.81
101	Fenamiphos sulfoxide	2.5	0.9981	0.5–50	2.5	112.5	2.1	50	93.3	0.5	4.81
102	Fenarimol	1	0.9982	1–50	1	91.2	16.6	50	98.6	1.6	1.5
103	Fenbuconazole	2.5	0.9983	0.5–50	2.5	72.9	7.9	50	99.3	4.2	1.24
104	Fenfuram	2.5	0.9983	0.5–50	2.5	78.7	4.6	50	100.8	1.1	−1.88
105	Fenobucarb	1	0.9979	0.5–50	1	95.7	3	50	88.3	6.4	24.28
106	Fenothiocarb	2.5	0.9993	0.5–50	2.5	106.5	8	50	98.6	0.9	−28.49
107	Fenoxanil	2.5	0.9983	0.5–50	2.5	82.6	8	50	106.3	0.7	−7.56
108	Fenoxycarb	2.5	0.9999	0.5–50	2.5	88.7	8.5	50	94.7	6.3	0.53
109	Fenpropimorph	1	0.9999	0.5–50	1	92.3	2.7	50	98	2.9	−24.4
110	Fenpyroximate	1	0.9994	0.5–50	1	91.6	2.5	50	99.1	3.1	−11.07
111	Fenthion	2.5	0.9993	0.5–50	2.5	98	6.4	50	94.9	7.4	−7.62
112	Fenthion oxon	1	0.999	0.5–50	1	88.4	5.2	50	101.5	0.7	−9.63
113	Fenthion oxon sulfone	2.5	0.9992	0.5–50	2.5	81.8	1.6	50	90.6	0.2	−34.61

114	Fenthion oxon sulfoxide	1	0.9999	1–50	1	109.5	10.9	50	91.9	4.8	−31.27
115	Fenthion sulfoxide	1	0.9991	0.5–50	1	85.7	8.4	50	96.2	2.9	−11.07
116	Fenthion-sulfone	2.5	0.9992	1–50	2.5	90.2	10.9	50	97.7	1.6	3.61
117	Ferimzone	1	0.9996	0.5–50	1	78.3	3.8	50	95.2	5.6	−2.07
118	Fipronil	2.5	0.9986	0.5–50	2.5	113.4	5.4	50	82.9	1.9	−0.62
119	Fipronil-sulfone	2.5	0.9986	0.5–50	2.5	86.1	12.1	50	95.7	0	−5.15
120	Flamprop-isopropyl	2.5	0.9992	0.5–50	2.5	106	2.7	50	93.4	4.7	−5.22
121	Flonicamid	1	0.9996	0.5–50	1	82.7	16.3	50	104.1	9.9	−3.71
122	Fluazinam	1	0.9998	0.5–50	1	106.8	1	50	92.8	0.2	−8.14
123	Flucetosulfuron	5	0.9995	0.5–50	5	79	0.4	50	77.6	6.5	18.58
124	Fludioxonil	2.5	0.9997	0.5–50	2.5	105	0.1	50	95.1	0.1	−18.86
125	Flufenacet	5	0.9963	1–50	5	89.8	8.6	50	84.1	10.1	−4.16
126	Flufenoxuron	1	0.9999	0.5–50	1	106.2	5.9	50	96.4	3.1	−0.43
127	Fluometuron	2.5	0.9995	2–50	2.5	73.5	2.7	50	89.6	3.8	−1.1
128	Fluopicolide	1	0.9999	0.5–50	1	100.8	2.3	50	102.1	0.5	9.99
129	Fluopyram	5	0.9954	0.5–50	5	90.4	3.5	50	98.7	2.3	1.2
130	Flupyradifuron	1	0.9995	0.5–50	1	75.2	11.4	50	87.9	5.5	−33.37
131	Fluquinconazole	2.5	0.9962	0.5–50	2.5	100.6	18.5	50	94.1	10.2	−7.53
132	Fluridone	1	0.9999	0.5–50	1	107.4	1.1	50	94.4	0.3	−18.87
133	Flurochloridone	2.5	0.9982	0.5–50	2.5	73.7	0.4	50	98.1	4.1	−6.63
134	Flurtamone	5	0.9924	0.5–50	5	78.6	17.5	50	95.7	2.5	0.11
135	Flusilazole	2.5	0.9988	0.5–50	2.5	79.5	3.6	50	88.7	3.5	−9.37
136	Fluthiacet-methyl	2.5	0.9997	0.5–50	2.5	87.6	3.3	50	101	1.9	−1.35
137	Flutianil	2.5	0.9998	0.5–50	2.5	96.9	3.1	50	99.9	4.3	−12.77
138	Flutolanil	1	0.9951	0.5–50	1	77.5	0.5	50	90.8	3.4	7.98
139	Flutriafol	1	1	0.5–50	1	77.6	3.5	50	89.7	1.9	−1.57
140	Fluxapyroxad	2.5	0.9988	0.5–50	2.5	85.8	8.8	50	102.7	3.5	9.12
141	Fosthiazate	1	0.9998	0.5–50	1	107.1	1.6	50	95.9	2.5	−9.45
142	Furathiocarb	1	0.9997	0.5–50	1	114.6	4	50	93.2	3.9	−1.07
143	Heptenophos	2.5	0.9984	0.5–50	2.5	107.9	3	50	106.4	12.9	14.45
144	Hexaflumuron	2.5	0.9981	0.5–50	2.5	72.4	16.7	50	93.9	2.1	−3.4
145	Hexazinone	2.5	0.997	1–50	2.5	82.4	6	50	98.6	0.6	−1.55
146	Hexythiazox	2.5	0.9982	0.5–50	2.5	85.6	4	50	93.8	5.8	−12.66
147	Imibenconazole	1	0.9999	0.5–50	1	98.5	17.3	50	89.5	8.1	1.38
148	Imicyafos	2.5	0.9951	0.5–50	2.5	113.2	3.3	50	89.3	2.7	−33.84
149	Imidacloprid	1	0.9993	0.5–50	1	97.6	3.9	50	90	3	0.48
150	Inabenfide	1	0.9997	0.5–50	1	94.5	0.8	50	95.7	2	−6.45
151	Indanofan	2.5	0.9991	0.5–50	2.5	115.8	8.6	50	90.3	2.5	10.05
152	Ipconazole	5	0.9943	0.5–50	5	90.4	16.9	50	94.9	8.7	2.52
153	Iprobenfos	2.5	0.9954	0.5–50	2.5	73.5	9	50	104.2	1.1	−27.73
154	Iprovalicarb	1	0.9994	0.5–50	1	97.8	16.5	50	96.1	10.3	−12.17
155	Isoprocarb	1	0.9981	0.5–50	1	111.8	12.2	50	98.2	3.9	10.47
156	Isoprothiolane	1	0.9996	0.5–50	1	86.5	7.6	50	97.6	3.8	−7.17
157	Isopyrazam	2.5	0.9991	1–50	2.5	84.1	2.8	50	91.1	6.4	−0.18
158	Isoxaben	5	0.9959	0.5–50	5	99.1	4.2	50	108.5	1.3	−7.73
159	Lenacil	2.5	0.9999	0.5–50	2.5	110.5	5.5	50	90.7	0.3	15.09
160	Linuron	5	0.9976	0.5–50	5	89.9	7.7	50	97.7	1.6	−1.56

161	Lufenuron	2.5	0.9999	0.5–50	2.5	94.4	0.2	50	80.2	17.2	1.54
162	Malaoxon	1	0.9998	0.5–50	1	116.3	11.9	50	98.5	3.1	−15.13
163	Mandipropamid	2.5	0.9996	0.5–50	2.5	91.9	17.9	50	94.8	2.1	−0.49
164	Mecarbam	2.5	0.9979	1–50	2.5	81.3	11.7	50	100.8	10.1	2.6
165	Mefenacet	2.5	0.9974	0.5–50	2.5	94.1	1.4	50	95.5	2.1	−9.13
166	Mepanipyrim	2.5	0.9983	0.5–50	2.5	88.6	0.3	50	92	13.5	0.98
167	Mephosfolan	1	0.9993	0.5–50	1	79	8.4	50	97.1	1.4	−28.93
168	Mepronil	5	0.9958	0.5–50	5	97	2.2	50	93.5	3.7	−5.42
169	Metaflumizone (1)	1	0.9959	1–50	1	82.6	11.2	50	96.1	10.4	−8.6
	Metaflumizone (2)	5	0.996	0.5–50	5	93	7.6	50	90.4	11	−8.6
170	Metalaxyl	2.5	0.9996	0.5–50	2.5	103.2	1.4	50	80.2	6.2	0.4
171	Metamifop	2.5	0.9975	0.5–50	2.5	72.1	0	50	101.2	12.2	−3.48
172	Metconazole	5	0.9992	0.5–50	5	89.4	5.1	50	96.1	1.6	1.53
173	Methabenzthiazuron	1	0.9999	0.5–50	1	109.5	5.2	50	95.9	1.6	−39.75
174	Methiocarb-sulfone	1	0.9998	0.5–50	1	104.6	9.5	50	92.7	1	−47.29
175	Methiocarb-sulfoxide	1	0.9994	0.5–50	1	85.7	4.9	50	96	0.5	−8.11
176	Methomyl	1	0.9993	0.5–50	1	87	15.6	50	98.4	0.4	−34.39
177	Methoprotryne	2.5	0.9995	0.5–50	2.5	96.6	2.7	50	100.6	2.7	−7.75
178	Metobromuron	1	0.9996	0.5–50	1	86.4	9.8	50	98.9	1.1	−0.23
179	Metominostrboin (Z)	1	0.9998	0.5–50	1	114.5	0.5	50	94.9	1.3	−6.49
180	Metominostrobin (E)	2.5	0.9961	0.5–50	2.5	72.8	2.2	50	97.4	2	−5.29
181	Metrafenon	2.5	0.9988	0.5–50	2.5	94.9	0.5	50	100.1	4.4	1.86
182	Mevinphos (1)	1	0.9973	0.5–50	1	88.7	6.3	50	97.7	1.2	0.44
	Mevinphos (2)	5	0.9984	0.5–50	5	94	3	50	102.5	1.5	−17.41
183	Monocrotophos	1	0.9997	0.5–50	1	81.4	3.8	50	95.9	6.2	−12.92
184	Myclobutanil	2.5	0.9984	1–50	2.5	93.7	18.3	50	95.8	11	−7.12
185	Napropamide	5	0.9955	0.5–50	5	100.2	10.9	50	104.4	7.7	−2.55
186	Neburon	10	0.9933	1–50	10	85.3	5.3	50	100.9	5.3	−24.55
187	Novaluron	5	0.9944	0.5–50	5	85	2.7	50	91.9	0.4	−9.42
188	Nuarimol	1	0.9999	0.5–50	1	100.9	0.2	50	100.4	0.9	−2.86
189	Ofurace	1	0.9999	0.5–50	1	94.8	5.4	50	90.5	3.5	−19.4
190	Omethoate	1	0.9993	0.5–50	1	100	9	50	91.2	8.2	−46.54
191	Oxadiazon	2.5	0.9988	0.5–50	2.5	119	2.6	50	96.7	0.6	−16.52
192	Oxamyl	2.5	0.9996	0.5–50	2.5	110.6	10.7	50	98.4	3.7	−38.7
193	Oxaziclomefone	5	0.9978	0.5–50	5	93.6	11.4	50	96.9	11.6	−0.82
194	Penconazole	2.5	0.9937	0.5–50	2.5	83	3.2	50	94.3	9.9	−3.04
195	Pencycuron	5	0.9915	0.5–50	5	92.3	1.1	50	104	1.6	−2.74
196	Phenthoate	1	0.9995	0.5–50	1	91.7	16.8	50	95.7	0.3	−33.72
197	Phosalone	2.5	0.9993	1–50	2.5	86.1	7.5	50	88.7	11.9	−6.44
198	Phosfolan	5	0.9983	0.5–50	5	104	2.5	50	96	0.4	−6.95
199	Phosmet-oxon	2.5	0.9984	0.5–50	2.5	77.9	2.1	50	100.9	0.2	−3.91
200	Phosphamidon	1	0.9998	0.5–50	1	115.2	5.1	50	98.3	0.7	1.45
201	Phoxim	2.5	0.9999	0.5–50	2.5	86.5	4.6	50	85.6	6.8	−15.19
202	Picolinafen	5	0.9955	1–50	5	94.4	15.1	50	103.6	8.8	−6.87
203	Pinoxaden	2.5	0.9989	0.5–50	2.5	84.5	2.8	50	93.5	3.4	1
204	Piperonyl butoxide	2.5	0.9973	0.5–50	2.5	78.4	1.4	50	101.3	1.4	−4.36
205	Piperophos	2.5	0.999	0.5–50	2.5	88.7	0.7	50	90.9	9.7	0.83

206	Pirimicarb	1	0.9998	0.5–50	1	75.9	9.8	50	97.1	0.8	−20.86
207	Pirimicarb-desmethyl	2.5	0.9991	0.5–50	2.5	102.9	1.1	50	85.2	3.7	−40.13
208	Pirimiphos-ethyl	5	0.9932	0.5–50	5	104.3	8.7	50	105.4	2.8	−7.23
209	Pirimiphos-methyl	1	0.9994	0.5–50	1	76.3	5	50	90.8	3.2	−3.05
210	Prochloraz	2.5	0.9985	0.5–50	2.5	103.2	16.1	50	98.8	3.4	−0.03
211	Profenofos	5	0.9989	0.5–50	5	91.1	11	50	89.8	13.9	−4.03
212	Promecarb	1	0.9998	0.5–50	1	98.3	7.8	50	97.8	3.2	2.26
213	Prometryn	1	0.994	1–50	1	78.9	17.9	50	95.7	3.7	9.63
214	Pronamide (Propyzamide)	5	0.9924	0.5–50	5	87.4	4.7	50	101.3	0.1	−0.36
215	Propachlor	2.5	0.9992	0.5–50	2.5	90.4	0.9	50	100.9	3.7	−9.21
216	Propamocarb	1	1	0.5–50	1	85.2	1.4	50	92.7	0.4	−32.29
217	Propanil	2.5	0.9982	1–50	2.5	77.4	4	50	93	2.5	−36.89
218	Propaquizafop	2.5	0.9985	0.5–50	2.5	79.8	5.5	50	98	5.3	−2.13
219	Propargite	1	0.9995	0.5–50	1	78	1.6	50	98.5	0.1	−37.43
220	Propazine	1	0.9997	0.5–50	1	93.9	0.1	50	100.8	1.3	−25.18
221	Propiconazole	5	0.9983	0.5–50	5	95.2	2.8	50	99.8	5.5	−8.65
222	Propoxur	1	0.9999	0.5–50	1	107.2	16.3	50	103	0.8	−16.85
223	Proqunazid	1	0.9999	0.5–50	1	103.4	5.1	50	99.5	3.4	−10.15
224	Prothioconazole	2.5	0.9987	0.5–50	2.5	87.4	13.6	50	113.9	17.7	19.71
225	Prothioconazole-desthio	2.5	0.9994	1–50	2.5	91.1	3.9	50	100.7	11.5	−2.45
226	Pydiflumetofen	1	0.9997	0.5–50	1	89.4	10.3	50	93	5.2	−8.5
227	Pyracarbolid	5	0.9965	2–50	5	91.1	0.3	50	101.4	4.1	−5.11
228	Pyraclofos	10	0.9937	0.5–50	10	93.8	6.8	50	105.4	6.8	−5.82
229	Pyraclonil	2.5	0.9995	0.5–50	2.5	89.2	2	50	100.7	1.2	−2.51
230	Pyraclostrobin	1	0.9998	0.5–50	1	78.7	4.7	50	90.4	4.4	0
231	Pyraflufen-ethyl	2.5	0.9976	0.5–50	2.5	81.1	2.6	50	96.3	5	−1.7
232	Pyraziflumid	2.5	0.999	0.5–50	2.5	90.8	8.8	50	98.5	6.2	−9.11
233	Pyrazolate	2.5	0.996	0.5–50	2.5	72.5	16	50	100.6	3	−2.83
234	Pyrazophos	2.5	0.9987	0.5–50	2.5	116.5	6.3	50	89.2	2.3	7.52
235	Pyributicarb	1	0.999	0.5–50	1	101.8	5.7	50	87.1	1.8	−8.91
236	Pyridaben	1	0.9999	0.5–50	1	100.4	11.3	50	92.6	2.7	−6.26
237	Pyridalyl	2.5	0.9987	0.5–50	2.5	91.8	3.1	50	118.8	4.5	17.03
238	Pyrifluquinazon	1	0.9997	0.5–50	1	83.2	19.8	50	92.5	3.7	11.9
239	Pyriftalid	2.5	0.9974	0.5–50	2.5	86.9	12.2	50	100.6	2.5	−5.9
240	Pyrimethanil	5	0.9987	0.5–50	5	100.1	5.7	50	92.5	0.1	−10.39
241	Pyrimidifen	5	0.9919	0.5–50	5	93.3	13.8	50	98.9	2.9	−4.86
242	Pyrimisulfan	2.5	0.9979	0.5–50	2.5	81.8	3.3	50	91.3	1.9	7.25
243	Pyriproxyfen	5	0.9948	0.5–50	5	92.7	13.5	50	96.6	10.8	−11.56
244	Pyroquilon	2.5	0.9998	0.5–50	2.5	88.6	4.2	50	89.2	0.7	−11.88
245	Quinalphos	5	0.9949	0.5–50	5	95.4	6.4	50	91.7	4.8	−10.52
246	Quizalofop-ethyl	10	0.9922	0.5–50	10	94.6	8.9	50	95.3	8.9	−7.53
247	Simazine	2.5	0.998	0.5–50	2.5	94.3	1.4	50	99.2	2.2	−12.7
248	Simetryn	1	0.9999	0.5–50	1	92.3	6.2	50	100.6	5.3	7.19
249	Spinetoram (J)	1	0.9993	0.5–50	1	95.5	7.8	50	93.5	9.2	−1.6
250	Spinetoram (L)	5	0.9967	0.5–50	5	98.1	7.9	50	94.5	10.7	−2.8
251	Spinosyn A	2.5	0.9988	0.5–50	2.5	70.9	3.3	50	90.8	10	−33.98
252	Spinosyn D	2.5	0.9996	0.5–50	2.5	89.3	9.2	50	88.2	17.3	−3.82

253	Spirodiclofen	2.5	0.9998	0.5–50	2.5	90.7	7.5	50	106.4	4.4	−40.83
254	Spirotetramat-ketohydroxy	5	0.9997	0.5–50	5	107.1	10.4	50	89.2	3.1	3.5
255	Spirotetramat-monohydroxy	2.5	0.9999	0.5–50	2.5	90.6	14	50	97.7	2.7	15.16
256	Spiroxamine	2.5	0.9995	0.5–50	2.5	83.7	1.3	50	98.2	1.4	−13.65
257	Sulfentrazone	5	0.9996	0.5–50	5	98.4	6.7	50	96	8.1	−18.38
258	Sulfotep	1	0.9999	0.5–50	1	76.9	13.6	50	85.5	4.1	−7.82
259	Sulfoxaflor	5	0.9999	1–50	5	81	10.1	50	95.5	0.6	23.35
260	Sulprofos	1	1	0.5–50	1	91.3	5.7	50	95	2.6	−13.85
261	TCMTB	1	0.9994	0.5–50	1	102.1	8.7	50	85.1	7.8	−10.25
262	Tebufenpyrad	2.5	0.9988	0.5–50	2.5	74.3	8.9	50	92.8	9.1	−12.54
263	Tebuthiuron	1	1	0.5–50	1	77.8	14.4	50	94.4	1.1	2.75
264	Tepraloxydim	2.5	0.9987	0.5–50	2.5	75.8	11.8	50	94.8	2.5	4.71
265	Terbuthylazine	2.5	0.9968	0.5–50	2.5	76.8	5.6	50	96.6	0.6	2.93
266	Terbutryn	5	0.9933	1–50	5	93.5	4.1	50	88	7.5	0.58
267	Tetrachlorvinphos	1	0.9994	0.5–50	1	104	0.9	50	100	3.9	−16.55
268	Tetraconazole	5	0.9991	0.5–50	5	88.8	14.1	50	107.1	9.7	3.75
269	Thenylchlor	5	0.9963	0.5–50	5	82.4	7.4	50	98.8	1.9	−19.83
270	Thiabendazole	1	1	0.5–50	1	102.2	7.2	50	96.7	2.5	−41.29
271	Thiacloprid	2.5	0.9968	0.5–50	2.5	78.4	7.3	50	96.8	0.3	−17.26
272	Thidiazuron	1	0.9994	0.5–50	1	116.8	0.6	50	88.3	0.3	−5.55
273	Thifluzamide	5	0.9985	0.5–50	5	90.4	13	50	85.9	8.4	−3.21
274	Thiobencarb	1	1	0.5–50	1	83.8	3.3	50	96.8	1.6	21.86
275	Thiodicarb	5	0.9984	0.5–50	5	97.7	7.8	50	92.2	4.3	−2.41
276	Thionazin	5	0.9996	0.5–50	5	102.4	6	50	102	4.5	13.75
277	Tiadinil	5	0.9943	0.5–50	5	99.3	3.6	50	82.7	6.7	−3.69
278	Tolfenpyrad	1	0.9998	1–50	1	72.8	1.7	50	92.4	5.6	−2.17
279	Triadimefon	1	0.9999	1–50	1	86.8	1.4	50	98.8	0.9	−29.58
280	Triazophos	2.5	0.9903	0.5–50	2.5	92.2	13.8	50	97.3	1.6	−1.11
281	Tricyclazole	2.5	0.9986	0.5–50	2.5	83.5	0.6	50	99.5	1.3	−9.68
282	Trifloxystrobin	2.5	0.9984	0.5–50	2.5	81.7	5.1	50	94.8	5.6	−5.24
283	Triflumizole	2.5	0.9986	0.5–50	2.5	79.8	2.2	50	94.6	7.8	−11.19
284	Triflumuron	2.5	0.9987	1–50	2.5	95.5	0.9	50	100.4	13.2	−3.15
285	Triticonazole	5	0.9978	0.5–50	5	92.8	4	50	100.7	6.8	−1.77
286	Vamidothion	1	0.9988	0.5–50	1	78.1	14.3	50	97.9	0.4	−31.02
287	Zoxamide	2.5	0.9979	0.5–50	2.5	97.4	8.5	50	95	11.1	−6.76
